# Variants in the *MS4A* cluster interact with soluble TREM2 expression on biomarkers of neuropathology

**DOI:** 10.1186/s13024-024-00727-7

**Published:** 2024-05-18

**Authors:** Rebecca L. Winfree, Emma Nolan, Logan Dumitrescu, Kaj Blennow, Henrik Zetterberg, Katherine A. Gifford, Kimberly R. Pechman, Mabel Seto, Vladislav A. Petyuk, Yanling Wang, Julie Schneider, David A. Bennett, Angela L. Jefferson, Timothy J. Hohman, the Alzheimer’s Disease Neuroimaging Initiative*

**Affiliations:** 1 Vanderbilt Memory and Alzheimer’s Center, Vanderbilt University Medical Center, Nashville, TN USA; 2 Pharmacology Department, Vanderbilt University Medical Center, Nashville, TN USA; 3 Department of Neurology, Vanderbilt University Medical Center, Nashville, TN USA; 4 Epidemiology Doctoral Program, School of Medicine, Vanderbilt University, Nashville, TN USA; 5 Vanderbilt Genetics Institute, Vanderbilt University Medical Center, Nashville, TN USA; 6 Department of Psychiatry and Neurochemistry, Institute of Neuroscience and Physiology, Sahlgrenska Academy, University of Gothenburg, 431 41 Mölndal, Sweden; 7 Clinical Neurochemistry Laboratory, Sahlgrenska University Hospital, Mölndal, Sweden; 8 Department of Neurodegenerative Disease, UCL Institute of Neurology, London, UK; 9 UK Dementia Research Institute at UCL, London, UK; 10 Hong Kong Center for Neurodegenerative Diseases, Clear Water Bay, Hong Kong, China; 11 Wisconsin Alzheimer’s Disease Research Center, University of Wisconsin School of Medicine and Public Health, University of Wisconsin-Madison, Madison, WI USA; 12 Biological Sciences Division, Pacific Northwest National Laboratory, Richland, WA USA; 13 Rush Alzheimer’s Disease Center, Rush University Medical Center, Chicago, IL USA; 14 Department of Pathology, Rush University Medical Center, Chicago, IL USA

**Keywords:** Alzheimer’s disease, MS4A, sTREM2, CSF biomarkers, Microglia, Inflammation, Blood–brain barrier

## Abstract

Recent evidence suggests that Alzheimer’s disease (AD) genetic risk variants (rs1582763 and rs6591561) of the *MS4A* locus are genome-wide significant regulators of soluble TREM2 levels such that the minor allele of the protective variant (rs1582763) is associated with higher sTREM2 and lower AD risk while the minor allele of (rs6591561) relates to lower sTREM2 and higher AD risk. Our group previously found that higher sTREM2 relates to higher Aβ_40_, worse blood–brain barrier (BBB) integrity (measured with the CSF/plasma albumin ratio), and higher CSF tau, suggesting strong associations with amyloid abundance and both BBB and neurodegeneration complicate interpretation. We expand on this work by leveraging these common variants as genetic tools to tune the interpretation of high CSF sTREM2, and by exploring the potential modifying role of these variants on the well-established associations between CSF sTREM2 as well as *TREM2* transcript levels in the brain with AD neuropathology. Biomarker analyses leveraged data from the Vanderbilt Memory & Aging Project (*n* = 127, age = 72 ± 6.43) and were replicated in the Alzheimer’s Disease Neuroimaging Initiative (*n* = 399, age = 73 ± 7.39). Autopsy analyses were performed leveraging data from the Religious Orders Study and Rush Memory and Aging Project (*n* = 577, age = 89 ± 6.46). We found that the protective variant rs1582763 attenuated the association between CSF sTREM2 and Aβ_40_ (β = -0.44, *p*-value = 0.017) and replicated this interaction in ADNI (β = -0.27, p = 0.017). We did not observe this same interaction effect between *TREM2* mRNA levels and Aβ peptides in brain (Aβ total β = -0.14, *p =* 0.629; Aβ_1-38_, β = 0.11, *p =* 0.200). In contrast to the effects on Aβ, the minor allele of this same variant seemed to enhance the association with blood–brain barrier dysfunction (β = 7.0e-4, *p =* 0.009), suggesting that elevated sTREM2 may carry a much different interpretation in carriers vs. non-carriers of this allele. When evaluating the risk variant (rs6591561) across datasets, we did not observe a statistically significant interaction against any outcome in VMAP and observed opposing directions of associations in ADNI and ROS/MAP on Aβ levels. Together, our results suggest that the protective effect of rs1582763 may act by decoupling the associations between sTREM2 and amyloid abundance, providing important mechanistic insight into sTREM2 changes and highlighting the need to incorporate genetic context into the analysis of sTREM2 levels, particularly if leveraged as a clinical biomarker of disease in the future.

**Supplementary Information:**

The online version contains supplementary material available at 10.1186/s13024-024-00727-7.

## Introduction

Rare variants associated with Alzheimer’s disease (AD) in the Triggering Receptor on Myeloid Cells 2 (*TREM2*) gene [1, 2], along with common variants in the membrane-spanning 4-domains subfamily A (*MS4A*) gene cluster, [3–8] have implicated the innate immune system in AD pathogenesis [9]. Interestingly, emerging genomic and functional evidence has suggested that these two genomic regions may interact or converge on a common disease pathway with myeloid lineage cells [10]. In the case of *TREM2*, numerous lines of functional evidence have implicated *TREM2* in AD pathogenesis [11] and also shaped the characterization of soluble TREM2 protein (sTREM2) as a biomarker in cerebrospinal fluid (CSF) [12, 13]. CSF sTREM2 levels appear to decrease with the onset of amyloidosis, and then rise with the onset of neurodegeneration [14, 15], likely as a biomarker of neuroinflammation in response to disease pathology.

Interestingly, since the initial discovery of common variants in the *MS4A* cluster associated with AD, two of those same variants have shown a robust association with CSF sTREM2 [10, 16]. The main signal, rs1582763, was associated with higher CSF sTREM2 expression and was previously linked to decreased risk of AD. In contrast, rs6591561 was associated with lower CSF sTREM2 levels, but increased risk of AD. Higher levels of CSF sTREM2 have indeed been associated with better cognitive and neuropathological outcomes in AD [17–21]. Genetic and functional studies indicate a role of MS4A4A and/or MS4A6A upstream of TREM2 and specifically link to changes in sTREM2 expression during AD pathogenesis [10, 16, 22]. Thus, the genetic variants in the *MS4A* cluster provide an exciting tool to better understand the complex changes in sTREM2 levels that occur over the AD cascade.

Changes in CSF sTREM2 levels over the course of AD are quite complex and have presented challenges in the development of sTREM2 as a biomarker of disease. Our group has previously characterized the biological correlates of elevated CSF sTREM2 during the preclinical phase of disease [15]. We found that high sTREM2 related to high CSF Aβ_40_ concentration (a soluble biomarker for amyloidogenic amyloid precursor protein processing), worse blood–brain barrier integrity (measured with the CSF/plasma albumin ratio), and higher CSF tau. Together, our results suggest that the interpretation of high CSF sTREM2 is confounded by its association with both Aβ abundance and neurodegeneration. Previously, we have shown that the strong association between CSF sTREM2 levels and high Aβ_40_ is important for longitudinal memory performance, and we are interested in whether this association is influenced by genetic regulation in the *MS4A* locus. We hypothesize that the genetic drivers of sTREM2 in the *MS4A* cluster may help us tune the interpretation of high CSF sTREM2 and increase its clinical utility. Specifically, we hypothesize that the protective variant, rs1582763 (MAF = 0.37), will attenuate the association between CSF sTREM2 levels and biomarkers of AD neuropathology, while the risk variant rs6591561 (MAF = 0.32) will amplify the association between CSF sTREM2 levels and biomarkers of AD neuropathology. Secondary analyses will also assess whether the *MS4A* variants modify the association between CSF sTREM2 and both BBB dysfunction and cognitive decline. Finally, given previously known associations between sTREM2 and TREM2 transcripts [23], we expand on the biomarker associations by looking at the modifying role of these common variants on associations between *TREM2* transcripts and AD-relevant outcomes in the brain.

## Methods

Participant data was acquired from four separate cohort studies of aging and dementia. The Vanderbilt Memory and Aging Project (VMAP) was utilized as a discovery cohort while the Religious Orders Study (ROS), Rush Memory and Aging Project (MAP), and the Alzheimer’s Disease Neuroimaging Initiative (ADNI) were utilized for replication analysis.

### VMAP cohort description

VMAP is a longitudinal study focused on the intersection between vascular health and brain aging [24]. Participants were 60–92 years of age including those with mild cognitive impairment (MCI) as determined by the National Institute on Aging/Alzheimer’s Association Workgroup [25]. The study also included age-, sex-, and race-matched cognitively normal controls (NC). This cohort includes participants with NC and MCI allowing for the characterization of biomarker changes within an early preclinical window when neuropathology begins to develop before the onset of clinical dementia. In summary, MCI diagnosis includes a Clinical Dementia Rating (CDR) score ≥ 0.5, concern of changes in cognition (reported by the participant, informant, or clinician), absence of dementia, relatively spared daily functioning, and neuropsychological functioning indicating objective impairment outside age-adjusted mean performance in one or more cognitive domains. Inclusion criteria required participants to speak English, have adequate auditory and visual acuity, and have a reliable study partner. Exclusion criteria included MRI contraindications, history of neurological disease or major psychiatric illness, heart failure, head injury with loss of consciousness > 5 min, or a systemic or terminal illness. All participants gave their written informed consent prior to data collection. Protocols were approved by the Vanderbilt University Medical Center Institutional Review Board.

### ROS/MAP cohorts description

Both cohorts enroll older participants without known dementia who agree to annual clinical evaluation and organ donation at death [26]. ROS enrolls older nuns, priests, and brothers from across the USA and MAP enrolls lay persons across the greater Chicago area. Both studies were approved by an Institutional Review Board of Rush University Medical Center and all participants signed informed and repository consents and an Anatomic Gift Act.

### ADNI cohort description

The Alzheimer’s Disease Neuroimaging Initiative (ADNI) is a large multicenter study enrolling participants with MCI, AD, and demographic-matched controls. The study design and detailed methods may be found at: https://adni.loni.usc.edu/. ADNI began in 2004 and is an ongoing global longitudinal study with 63 sites in the U.S. and Canada.

### Lumbar puncture and biochemical analyses

Briefly, an optional baseline, fasting, lumbar puncture procedure aided CSF collection using a polypropylene syringe and a Sprotte 25-gauge spinal needle from an intervertebral lumbar space. CSF supernatant was immediately extracted and stored at -80 °C. Commercially available kits were used for the measurement of CSF total tau, p-tau181, Aβ_x-40_, Aβ_x-42_, Aβ_1-42_, neurofilament light (NfL) and albumin concentrations, performed in batch, as previously described. Albumin was measured in both CSF and plasma by immunonephelometry [15]. A Meso Scale Discovery (MSD) assay (Rockville, MD) was used to measure sTREM2 levels in CSF, described by Jensen et al., 2019 [27]. Commercially available enzyme-linked immunosorbent assays (Fujirebio, Ghent, Belgium) were used to determine the levels of Aβ_42_ (INNOTEST β-AMYLOID(1–42)) and the subsequent Aβ_42_/Aβ_40_ ratio [24]. Biotinylated polyclonal goat anti-human TREM2 capture antibodies (R&D Systems BAF1828) were incubated with recombinant human TREM2 protein (Sino Biological Inc. 11084-H08H) and a SULFO-TAG–labeled anti-mouse secondary antibody was used for detection. More detailed kit information is provided in Supplemental Table 1.

### Neuropsychological composites

Neuropsychological assessments of cognitive performance at baseline aided in the calculation of episodic memory and executive functioning composite z-scores. Details are previously described [15, 24]. The VMAP baseline memory score was calculated as a composite resulting from a z-score from the following tests: California Verbal Learning Test Second Edition (CVLT-II) Total Immediate Recall, CVLT-II Delayed Recall, CVLT-II Recognition, Biber Figure Learning Test (BFLT) Total Immediate Recall, BFLT Delayed Recall, and BLFT Recognition [28].

### Genotyping and quality control

Genotyping was performed using whole blood (VMAP and ADNI) or brain tissue extracted DNA (ROS/MAP) on the following genotyping arrays: Illumina MEGA^EX^ (VMAP); three Illumina platforms were used in ADNI: Human610-Quad, HumanOmniExpress, and Omni 2.5 M. ROS/MAP genotypes were also obtained on multiple platforms: Affymetrix Genechip 6.0, Illumina Human1M, and Illumina Global Screening Array. Sample sets genotyped on different arrays were processed and imputed in parallel and merged after imputation.

Standard QC was performed in accordance with the Computational Neurogenomics GWAS pipeline (Vanderbilt Memory and Alzheimer’s Center) which excluded variants with genotyping efficiency < 95% or minor allele frequency (MAF) < 1. Additionally, samples with call rates < 99%, who exhibited an inconsistency between reported and genetic sex (*i.e.*, assigned females with an X chromosome homozygosity estimate > 0.8 or assigned males with a value < 0.2), who displayed excess relatedness (*i.e.*, pi-hat > 0.25) or excessively high or low rates of heterozygosity (*i.e.*, > 5 SD) were removed. In order to detect samples with large-scale differences in ancestry, principal components (PCs) were calculated using 1000 Genomes (http://ftp.1000genomes.ebi.ac.uk/vol1/ftp/release/20130502/) as reference populations. Individuals who self-reported as non-Hispanic White were kept. PLINK, versions 1.9 and 2.0 (https://www.cog-genomics.org/plink/) were used. Prior to imputation, all palindromic, multi-allelic or duplicated variants were removed. Then, variant positions were lifted over to genome build 38 (when appropriate) and compared to the TOPMed reference panel. Variant strand, position, and reference allele assignment were updated, where necessary. Variants were excluded if they failed lift-over or did not match the reference panel.

Imputation was performed on the TOPMed Imputation Server version 1.5.7 (https://imputation.biodatacatalyst.nhlbi.nih.gov/), using Minimac4, and Eagle for phasing. The genetic data was then filtered for imputation quality (*R*^2^ > 0.8) and multiallelic SNPs. Finally, variants were filtered for MAF > 0.01, and Hardy–Weinberg Equilibrium tested by a χ2 goodness of fit between observed and expected genotypes at a threshold of *p* > 1E-06. Genetic ancestry was assessed using PCs and sample outliers were identified and removed.

### ADNI CSF Aβ species and sTREM2 measurement

ADNI cohort demographics (*N* = 440) are provided in Supplemental Table 2. Baseline CSF measurements of Aβ_1-42_, Aβ_1-40_, and Aβ_1-38_ were acquired utilizing 2D-UPLC tandem mass spectrometry. Each data point represents the average of duplicate 0.1 mL aliquots from a single CSF sample. Sample processing and detailed methodology is described elsewhere [29] and is publicly available for download on the ADNI database (adni.loni.usc.edu). An in-house assay quantified CSF sTREM2 and has been previously established and validated [12, 14, 30, 31].

### ROS/MAP SRM proteomic measures of Aβ and Tau

ROS/MAP cohort demographics are provided in Supplemental Table 3 describing (*N* = 577) individuals with measurements of *TREM2* mRNA and Aβ and tau peptides. Frozen dorsolateral prefrontal cortex (dlPFC) tissue samples were utilized for single reaction monitoring mass spectrometry (SRM) proteomic analysis. Sample preparation and standard protocols are previously described [32, 33]. Experiments were carried out using a nano ACQUITY UPLC coupled to TSQ Vantage MS instrument where 2uL of sample was injected for each measurement. Peptide separations were performed by an ACQUITY UPLC BEH 1.7 µm C18 column (75 µm i.d. × 25 cm) at a flow rate of 350nL/min. The heated capillary temperature and spray voltage was set at 350 °C and 2.4 kV, respectively. Both the Q1 and Q3 were set as 0.7FWHM (full width at half maximum). A scan width of 0.002 m/z and a dwell time of 10 ms were used. Data were analyzed by Skyline software [34]. Accuracy of peak assignments and boundaries were inspected manually. The peak area ratios of endogenous light peptides and their labeled internal standards were automatically calculated by Skyline. Endogenous peptide quantification was assessed as a ratio of spiked-in synthetic heavy isotope-labeled peptides (“light”/”heavy”). Relative abundance of each peptide was normalized yielding a median log2-transformed ratio of zero to adjust for differences in protein expression between samples. Quality control of signal to noise ratio was assessed as the ratio of variance across subject samples to that of technical controls. This involved a scattering of pooled control samples throughout the experiment (8 samples/96-well plate) allowing for capture of technical variance from sample preparation or instrumental measurement. Peptide measurements with a signal to noise ratio of less than 2, > 10% missingness and/or > 4 standard deviations from the mean were removed prior to model analyses.

### Statistical analysis

Statistical analyses were performed in R v3.6.1 using R Studio IDE (https://www.rstudio.com/). To evaluate the data, multiple linear regression models (for cross-sectional cognition and AD-related pathology outcomes) were used. Linear regression models covaried for age (or age at death), participant sex, post-mortem interval (when appropriate), and education. Models assessed *MS4A* SNP interactions with CSF sTREM2 protein or *TREM2* mRNA expression levels on the following AD clinical and neuropathological outcome measures: cross-sectional baseline cognition (VMAP/ADNI), CSF biomarkers Aβ_1-42_, Aβ_x-42_, Aβ_x-40_, p-tau, t-tau, CSF/plasma albumin ratio (VMAP), mass spectrometry measurement of Aβ_1-42_, Aβ_1-40_, Aβ_1-38_, (ADNI), and mass spectrometry measurement (SRM) of tau AT8, total Aβ, and Aβ_1-38_ peptides (ROS/MAP). ADNI and ROS/MAP data allowed for replication of SNP interactions from initial findings using the VMAP discovery cohort including outcome measures of cognition, tau, and Aβ. For example, interaction models took the general form: [AD biomarker expression ~ TREM2 expression X presence of MS4A SNP + demographic covariates], where AD biomarker expression is the outcome and TREM2 expression (CSF of transcript) and one of the two MS4A SNPs were the explanatory variables of interest. All interaction models included all lower-order main effects.

Sensitivity analyses assessed potential variability in results due to vascular comorbidity excluding seven participants with probable cerebral amyloid angiopathy in VMAP based on elevated microbleed count. These models also included the Framingham Stroke Risk Profile Score [24] as a covariate and yielded comparable results (Supplemental Table 4).

All models were corrected for multiple comparisons using the Benjamini & Hochberg [35] false discovery rate based on the total number of tests completed, accounting for all interaction analyses. Statistical outliers of *TREM2* mRNA or sTREM2 protein outside four standard deviations from the mean were removed.

## Results

### Participant demographics

A discovery cohort of non-Hispanic White individuals with CSF sTREM2 measurement was leveraged from the Vanderbilt Memory and Aging Project (VMAP). *MS4A*-associated SNP interaction analyses included data from individuals with sTREM2 measurement and genotype information for rs1582763 and rs6591561 (*N* = 127). This cohort did not include carriers of *TREM2* rare variants, R47H, R62H, D87N, or H157Y. The ROS/MAP and ADNI cohorts did include a handful of variant carriers. However, all results remained when removing these 16 individuals from our analyses (Supplemental Tables 5–7). Participants diagnosed with MCI had fewer years of education than those with NC (*p =* 0.003; Table 1). A sample size breakdown of *MS4A* carriers vs. non-carriers is provided in Supplementary Table 8. Main effects of the variants are presented in Supplemental Tables 9 and 10.

**Table 1:** VMAP cohort demographics

Characteristic	Clinical Diagnosis		Total Total (N=127)	*P*-value
	Normal Cognition (N=68)	Mild Cognitive Impairment (N=59)		
Male, no. (%)	48 (71)	39 (66)	87 (69)	0.725
Age (baseline)	72±6.69	72±6.15	72±6.43	0.512
Education (years)	17±2.42	15±2.92	16±2.75	**0.003**
*APOE-*ε4 carriers, no. (%)	19 (30)	23 (39)	42 (33)	0.259
sTREM2 CSF pg/mL	3572±1833.11	3844±1743.51	3699±1790.16	0.393

Values are presented as mean±standard deviation, unless otherwise indicated. A Student’s t-test or a Pearson’s chi-squared test was used to compare continuous or categorical variables, respectively, between cognitive diagnoses

Bold represents statistical significance set to a priori threshold *P*<0.05

### Minor allele of rs1582763 is associated with increased CSF sTREM2 levels

**Fig. 1:**
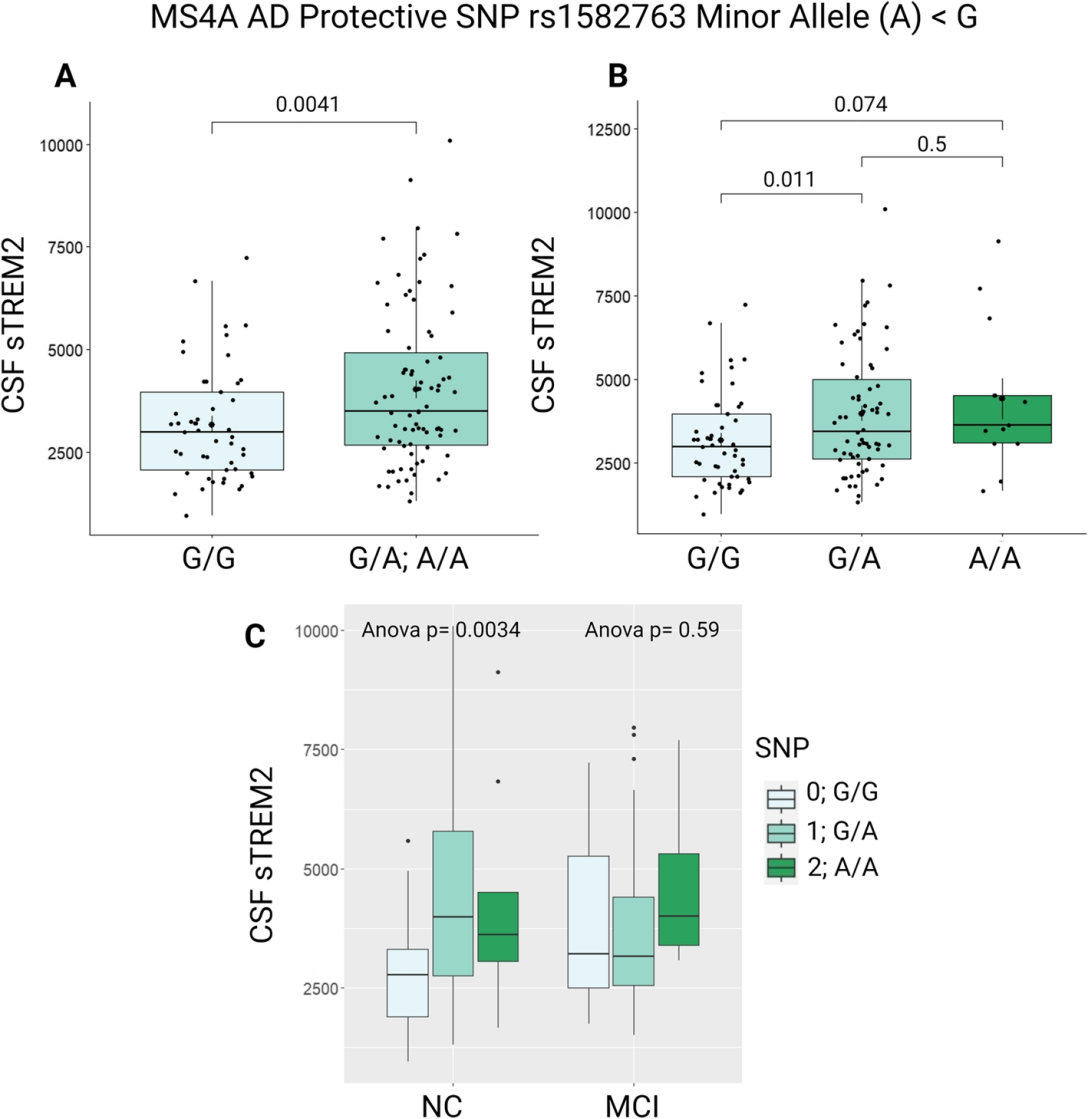
VMAP CSF sTREM2 characterization across rs1582763 genotype. **A** CSF sTREM2 levels are increased in minor allele (A) carriers compared to non-carriers (**B**) Increases in CSF sTREM2 are seen in heterozygote carriers compared to non-carriers. *P*-values reported by a student’s t-test. **C** sTREM2 expression changes according to the presence of minor allele by cognitive diagnosis. sTREM2 measurement in pg/mL. G/G; *N* = 49. G/A; *N* = 63. A/A; *N* = 15. Measurements in pg/mL

As expected, individuals carrying the minor allele (A < G) of rs1582763 had higher concentrations of sTREM2 in CSF as compared to non-carriers (Fig. 1A; *p =* 0.004). There was a dose-dependent response of CSF sTREM2 concentration to minor allele dosage. However, few individuals in this cohort were homozygous for rs1582763_A (Fig. 1B; *N* = 15). Additionally, these increased levels of sTREM2 in minor allele carriers were not specific to cognitive diagnosis, suggesting this SNP affects sTREM2 irrespective of cognitive impairment (Fig. 1C).

**Fig. 2:**
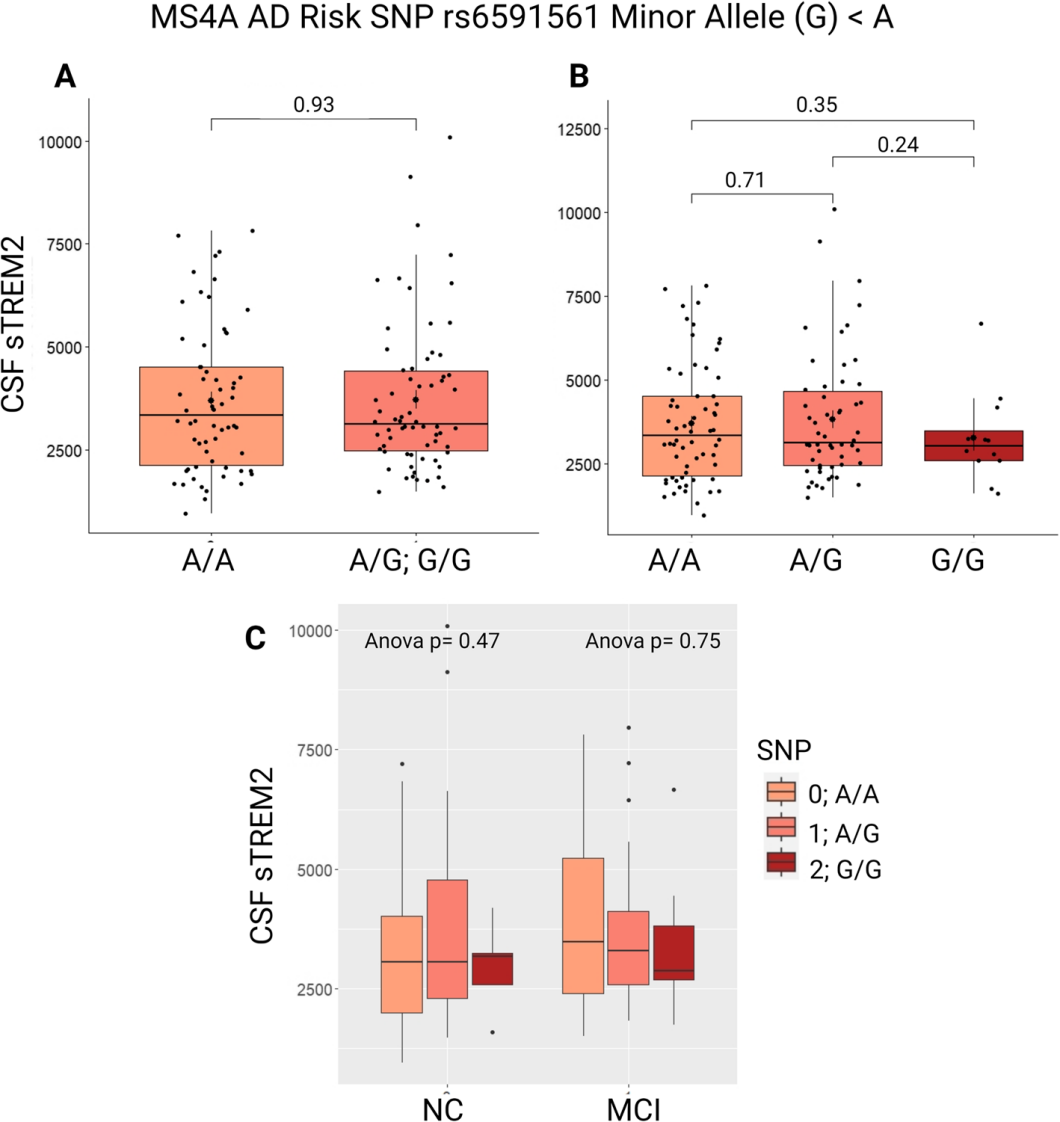
VMAP CSF sTREM2 characterization across rs6591561 genotype. **A**, **B** sTREM2 levels were not associated with rs6591561 genotype. *P*-values reported by a student’s t-test. **C** sTREM2 levels were not associated with rs6591561 genotype when stratified by cognitive diagnosis. sTREM2 measurement in pg/mL. A/A; *N* = 62. A/G; *N* = 54. G/G; *N* = 12. Measurements in pg/mL

In contrast to rs1582763 minor allele carriers, there was no association between sTREM2 levels and rs6591561 genotype (Fig. 2). We expected this risk allele, rs6591561_G, to be associated with decreased CSF sTREM2, supporting results from Deming et al. 2019 [10]. The lack of significant association may be due to our smaller sample size (*N* = 127) as compared to the initial report concerning 813 individuals in ADNI. Furthermore, individuals in ADNI included those with clinical AD diagnosis, whereas the present cohort did not include AD participants. The lack of association between sTREM2 levels and rs6591561 genotype remained across cognitive diagnosis of NC and MCI (Fig. 2C).

### rs1582763 minor allele interacts with CSF sTREM2 levels on select AD biomarkers of neuropathology

**Fig. 3:**
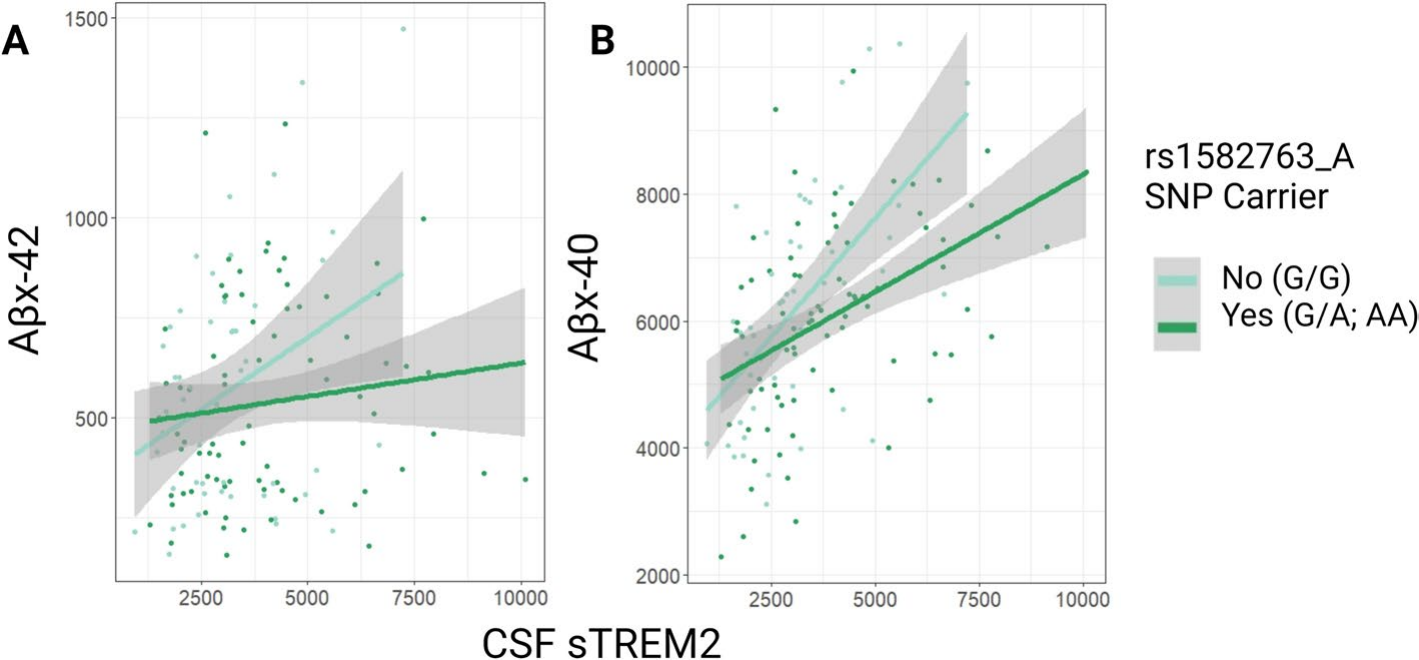
VMAP rs1582763 X sTREM2 interactions on Aβ peptides. Genetic regulation of MS4A (presence of rs1582763_A) attenuates the associations between increased sTREM2 and increased shorter Aβ peptide in CSF including (**A**) Aβ_x-42_ (pg/mL) β = -0.09, *p =* 6.0e-3 and (**B**) Aβ_x-40_ (pg/mL) β = -0.44, *p =* 1.7e-2. Measurements in pg/mL. Statistical results were determined by cross-sectional baseline linear regression models

**Fig. 4:**
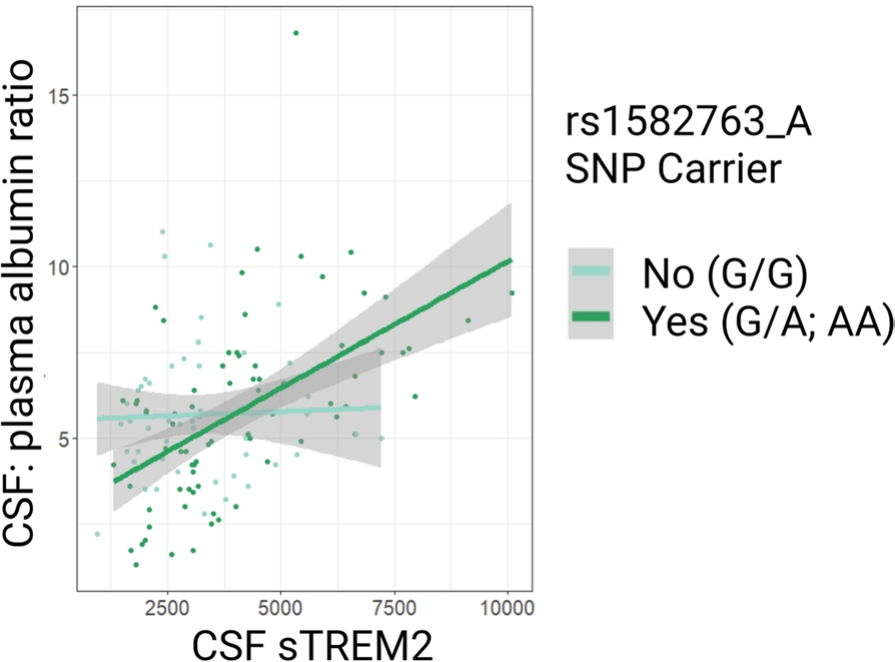
VMAP rs1582763 X sTREM2 interaction on CSF/plasma albumin. Genetic regulation of MS4A (presence of rs1582763_A) drives the association between increased CSF sTREM2 (pg/mL) and increased CSF/plasma Albumin ratio (β = 7.0e-4, *p =* 9.0e-3). Statistical results were determined by cross-sectional baseline linear regression models

Table 2 compiles interaction results (rs1582763 minor allele presence*CSF sTREM2 predicting neuropathology) from cross-sectional linear regression models across AD biomarkers of interest. As hypothesized, rs1582763_A modified the association between sTREM2 levels and shorter Aβ species in CSF. Specifically, the presence of rs1582763 minor allele attenuated the positive association between high sTREM2 and high N-truncated species (Aβ_x-42_) as well as Aβ_x-40_ in CSF (Fig. 3; β = -0.093, *p =* 0.006 and β = -0.439, *p =* 0.017, respectively). In contrast, we observed insignificant results with Aβ_1-42_ (Table 2; β = -0.051, *p =* 0.101) in keeping with a lack of main effect of CSF sTREM2 levels on this particular outcome in VMAP, which we have previously published [15]. Interestingly, the positive association between CSF levels of sTREM2 and a marker of increased BBB permeability, CSF/plasma albumin ratio, was driven by rs1582763_A carriers and absent in non-carriers (Table 2 and Fig. 4; β = 0.0007, *p =* 0.009). Finally, interaction results revealed that rs1582763_A did not significantly modify the positive association between sTREM2 levels and biomarkers of tau pathology in CSF (Table 2; t-tau, β = -0.036, *p =* 0.108; tau_181P_, β = -0.004, *p =* 0.114).

**Table 2:** sTREM2 * Presence of rs1582763 Minor Allele (A<G) Interaction on CSF AD biomarkers

VMAP Outcome	β	*P*
Aβ_x-42_	-0.093	0.006*
CSF/plasma Albumin ratio	0.0007	0.009*
Aβ_x-40_	-0.439	0.017*
t-Tau	-0.036	0.108
Aβ_1-42_	-0.051	0.101
Tau_181P_	-0.004	0.114

Cross-sectional linear regression models assessed interactions of sTREM2 CSF protein levels and the presence of MS4A variant, rs1582763, on VMAP CSF AD biomarker outcomes as quantified by ELISA

Bold represents statistical significance set to a priori threshold *P* < 0.05

^*^Signifies significance after multiple corrections using the Benjamini & Hochberg [35] false discovery rate based on number of tests completed

Secondary analysis revealed there was a weak interaction of sTREM2 and the presence of rs1582763_A on the memory composite (Supplemental Fig. 1A.; β = 2.35e-4, se = 1.14e-4, *p =* 0.042) whereas higher sTREM2 concentration was associated with worse cognitive performance in non-carriers of rs1582763_A. However, this trend was not significant using ADNI memory scores (Supplemental Fig. 1B).

### rs6591561 minor allele does not interact with CSF sTREM2 levels on AD biomarkers of neuropathology

In addition to a lack of association with CSF sTREM2 expression levels in VMAP, the presence of rs6591561 minor allele did not interact with sTREM2 expression on any biomarker of neuropathology. Table 3 summarizes these results including a lack of effect on N-truncated Aβ_x-42_ as well as Aβ_x-40_ (Supplemental Fig. 2A, B; β = -0.008, *p =* 0.757 and β = 0.112, *p =* 0.445, respectively). Similarly, interaction results for CSF/plasma albumin ratio were insignificant (Table 3; β = -2.190e^−4^, *p =* 0.303). The positive association between sTREM2 levels and this BBB integrity marker holds amongst both carriers and non-carriers of rs6591561 minor allele (Supplemental Fig. 2C).

**Table 3:** sTREM2 * Presence of rs6591561 Minor Allele (G<A) Interaction on CSF AD biomarkers

VMAP Outcome	β	*P*
Aβ_x-42_	-0.008	0.757
CSF/plasma albumin ratio	-2.190e^-4^	0.303
Aβ_x-40_	0.112	0.445
t-Tau	0.011	0.545
Aβ_1-42_	-0.020	0.411
Tau_181P_	0.001	0.552

Cross-sectional linear regression models assessed interactions of sTREM2 CSF protein levels and the presence of MS4A variant, rs6591561, on VMAP CSF AD biomarker outcomes as quantified by ELISA

Statistical significance set to a priori threshold *P* < 0.05

### ADNI replication results

Next, we sought to replicate these novel biomarker interaction results in an independent cohort. ADNI participants with available genotype, CSF sTREM2, and Aβ peptide tandem UPLC mass spectrometry measurements were leveraged yielding a replication cohort of *N* = 399. The demographic characteristics of this cohort are described in Supplemental Table 2.

**Fig. 5:**
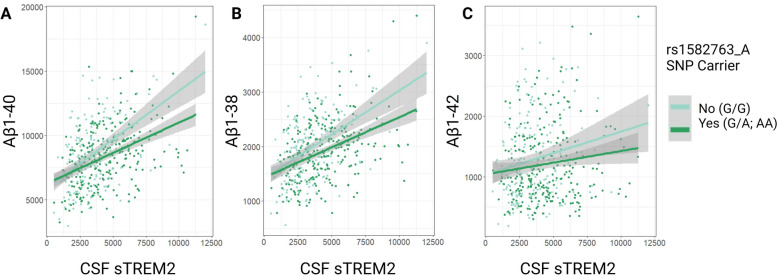
rs1582763 X sTREM2 interaction on Aβ peptides ADNI replication. The minor allele of rs1582763 significantly attenuates the positive association between sTREM2 and Aβ_1-40_ peptide levels in CSF in ADNI (β = -0.27, *p =* 0.02) (**A**). This attenuation is trending but non-significant for both (**B**) Aβ_1-38_ (β = -0.05, *p =* 0.09) and **C** Aβ_1-42_ (β = -0.03, *p =* 0.40). Measurements in pg/mL

The minor allele of AD protective SNP, rs1582763, interacted with sTREM2 levels on peptide measurements of Aβ_1-40_ (Table 4 and Fig. 5A; β = -0.269, *p =* 0.017), whereby the positive association between sTREM2 and Aβ_1-40_ expression was attenuated among carriers of rs1582763_A. Similarly, minor allele carriers had an attenuated association between sTREM2 and Aβ_1-38_ expression, albeit this interaction did not reach statistical significance (Table 4 and Fig. 5B; β = -0.045, *p =* 0.094). In accordance with previously published main effects results [15], the presence of rs1582763_A did not interact with sTREM2 levels on full-length Aβ_1-42_.

**Table 4:** sTREM2 * Presence of rs1582763 Minor Allele (A<G) Interaction on CSF AD biomarkers in ADNI

ADNI Outcome	β	*P*
Aβ_1-40_	-0.269	0.017*
Aβ_1-38_	-0.045	0.094
Aβ_1-42_	-0.027	0.396

Cross-sectional linear regression models assessed interactions of sTREM2 CSF protein levels and the presence of MS4A variant, rs1582763, on ADNI CSF amyloid biomarker outcomes as quantified by mass spectrometry

Bold represents statistical significance set to a priori threshold *P* < 0.05

^*^Signifies significance after multiple corrections using the Benjamini & Hochberg [35] false discovery rate based on number of tests completed

**Fig. 6:**
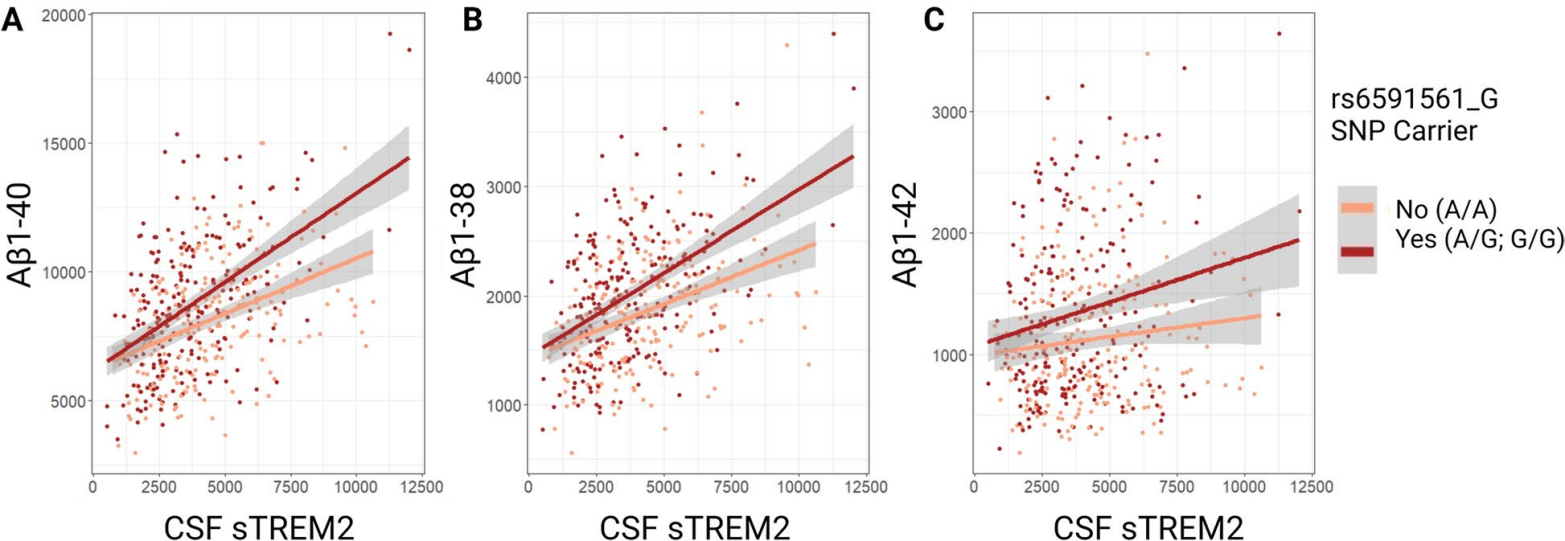
rs6591561 X sTREM2 interactions on Aβ peptides in ADNI. The minor allele (G) of rs6591561 is associated with a more positive association between sTREM2 and Aβ_1-40_ peptide levels in CSF in ADNI (β = 0.2, *p =* 0.03) (**A**). This effect is trending but non-significant for both (**B**) Aβ_1-38_ (β = 0.05, *p =* 0.05) and **C** Aβ_1-42_ (β = 0.09 = 2, *p =* 0.45). Measurements in pg/mL

In contrast to insignificant results using VMAP data for AD risk SNP rs6591561, in ADNI, the minor allele (G) interacted with CSF sTREM2 levels on Aβ_1-40_ expression whereby non-carriers of rs6591561_G had an attenuated association between sTREM2 and Aβ_1-40_ levels (Table 5 and Fig. 6A; β = 0.223, *p =* 0.028). Notably, the direction of this effect opposes those of rs1582763_A on shorter Aβ species as hypothesized. However, without significant independent replication between the datasets in our analyses, this novel association remains largely uninterpretable. In keeping with trends across Aβ peptide outcome measures, Aβ_1-38_ and Aβ_1-42_ exhibited similar results in terms of direction and magnitude with our discovery cohort (Table 5 and Fig. 6B, C; β = 0.046, *p =* 0.054 and β = 0.022, *p =* 0.454, respectively).

**Table 5:** sTREM2 * Presence of rs6591561 Minor Allele (G<A) Interaction on CSF AD biomarkers in ADNI

ADNI Outcome	β	*P*
Aβ_1-40_	0.223	0.028*
Aβ_1-38_	0.046	0.054
Aβ_1-42_	0.022	0.454

Cross-sectional linear regression models assessed interactions of sTREM2 CSF protein levels and the presence of MS4A variant, rs6591561, on ADNI CSF amyloid biomarker outcomes as quantified by mass spectrometry

Bold represents statistical significance set to a priori threshold *P* < 0.05

^*^Signifies significance after multiple corrections using the Benjamini & Hochberg [35] false discovery rate based on number of tests completed

### MS4A SNP interactions with TREM2 transcript expression on Aβ Levels at autopsy

**Fig. 7:**
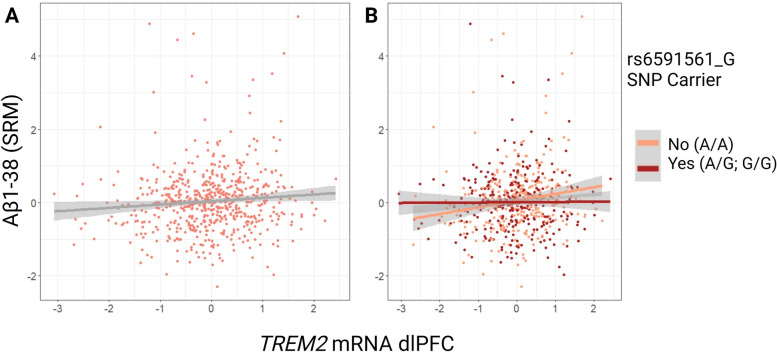
*TREM2* X rs6591561 interaction on Aβ_1-38_ in ROS/ MAP. **A** *TREM2* mRNA expression from the dorsolateral prefrontal cortex (dlPFC) is positively associated with Aβ_1-38_ as measured by single reaction monitoring mass spectrometry (SRM) β = 0.09, *p =* 0.03. **B** *TREM2* dlPFC mRNA expression association with Aβ_1-38_ is driven by non-carriers of rs6591561 G minor allele (β = -0.20, *p =* 0.02)

Next, we asked whether *MS4A* SNP interactions were coupled to *TREM2* transcript abundance using ROS/MAP data (Supplemental Table 3). Due to the lack of sTREM2 CSF data in ROS/MAP, we instead chose this cohort to determine whether *MS4A* genetic regulation of sTREM2 potentially generalizes to *TREM2* transcript levels and whether the intersection of these two pathways on neuropathology may be present post-mortem. The main effect of *TREM2* mRNA expression on Aβ neuropathological measurements in ROS/MAP are provided in Table 6. Using bulk RNA sequencing of *TREM2* mRNA and SRM-quantified measurements of Aβ species, we uncovered novel associations. First, the presence of rs6591561 minor allele predicted lower levels of *TREM2* mRNA from dorsolateral prefrontal cortex (dlPFC), (Table 8; β = -0.122, se = 0.054, *p =* 0.024). Second, there was an interaction of rs6591561 minor allele with dlPFC *TREM2* mRNA on Aβ_1-38_ measured by mass spectrometry from dlPFC tissue (Table 7 and Fig. 7B; β = 0.158, *p =* 0.003). The minor allele of rs1582763 was not associated with *TREM2* mRNA levels (Table 8; β = 0.055, se = 0.070, *p =* 0.430) and did not interact significantly on Aβ outcome measures.

**Table 6:** Main Effects of TREM2 mRNA on Aβ Neuropathology

Neuropathology Outcome Measure	Estimate	*P* Value
Aβ_1-38_ (SRM)	0.0921	0.031*
Aβ (total) (SRM)	0.484	1.62e-03*

Cross-sectional linear regression models assessed main effects of TREM2 mRNA levels on ROS/MAP Aβ neuropathology as quantified by SRM proteomic analysis

Bold represents statistical significance set to a priori threshold *P* < 0.05

^*^Signifies significance after multiple corrections using the Benjamini & Hochberg [35] false discovery rate based on number of tests completed

**Table 7:** ROS/MAP Interaction Effects of *TREM2* mRNA*SNP on Aβ Neuropathology

Neuropathology Outcome Measure	*rs1582763*		*rs6591561*	
	Estimate	P.int.Value	Estimate	P.int.Value
Aβ_1-38_ (SRM)	0.108	0.200	-0.203	0.016*
Aβ (total) (SRM)	-0.143	0.629	0.045	0.882

Cross-sectional linear regression models assessed interactions of TREM2 mRNA levels and the presence of MS4A variants, on ROS/MAP Aβ neuropathology as quantified by SRM proteomic analysis

Bold represents statistical significance set to a priori threshold *P* < 0.05

^*^Signifies significance after multiple corrections using the Benjamini & Hochberg [35] false discovery rate based on number of tests completed

Table 4.7. MS4A SNP * *TREM2* mRNA Interaction on Aβ Neuropathology

**Table 8:** Main Effects of *MS4A* SNPs on *TREM2* mRNA levels

*MS4A* SNP	Estimate	*P* Value
rs1582763	0.055	0.430
rs6591561	-0.122	**0.024**

Cross-sectional linear regression models assessed main effects of MS4A SNP status on ROS/MAP TREM2 mRNA levels at autopsy in the dorsolateral prefrontal cortex

Bold represents statistical significance set to a priori threshold *P* < 0.05

Additional analysis, utilizing SRM-quantified AT8 tau peptide as an outcome measure in ROS/MAP yielded insignificant main effect and SNP interaction results (Supplemental Tables 11 and 12).

## Discussion

Our work previously demonstrated that the association between higher sTREM2 and higher Aβ was relevant to late-life cognitive performance, and we speculated that the association may reflect a compensatory alteration in Aβ abundance in response to glial activation and/or altered neuronal activity [15]. The present analyses assessed whether *MS4A* AD-associated SNPs modify previously published associations between CSF sTREM2 levels and AD biomarkers of neuropathology [15]. In summary, we find that genetic regulation at the *MS4A* locus selectively modifies sTREM2 associations with multiple biomarkers of shorter Aβ species and BBB integrity but not those of tau. Moreover, in the brain, *MS4A* risk variant rs6591561_G modifies *TREM2* transcript associations with Aβ_1-38_ species. Together, results highlight several biological pathways through which these variants (rs1582763_A and rs6591561_G) may regulate AD resilience and risk. Results bolster support for the current theory that the TREM2 pathway plays a causal role in AD pathogenesis via inflammatory regulation of neuropathology. Importantly, a novel relationship between sTREM2 levels in CSF and BBB integrity in carriers which is absent in non-carriers of protective rs1582763_A may suggest that MS4A and TREM2 proteins function at the intersection of peripheral-central inflammatory communication.

### *MS4A* protective allele corresponds to increased CSF sTREM2 level

The association previously described in the literature between *MS4A* AD-protective rs1582763_A and CSF sTREM2 levels was recapitulated in the VMAP discovery cohort while the rs6591561_G association did not replicate (Fig. 2). Previously described decreases in sTREM2 associated with rs6591561_G risk allele were not observed in VMAP, we suspect, due to a much smaller sample size. As expected, protective SNP rs1582763_A was associated with elevated levels of sTREM2 in both VMAP and ADNI whereas rs6591561_G was associated with decreased sTREM2 only in ADNI. This increases confidence that sTREM2 production is genetically regulated by the *MS4A* locus. The relationship between genotype and sTREM2 protein abundance in CSF was true across cognitive diagnosis indicating genetic regulation by the *MS4A* region of sTREM2 production is likely a constitutive as opposed to a disease-specific process.

### The *MS4A* protective variant attenuates the association between sTREM2 and the abundance of shorter Aβ species, while the *MS4A* risk variant enhances such associations

The main interaction findings from the VMAP discovery cohort demonstrate the presence of protective rs1582763 minor allele modified previously established main effects between sTREM2 and Aβ_x-40_, and the relationship between sTREM2 and Aβ_x-42_ (Table 2). Genetic regulation by protective *MS4A* rs1582763_A attenuates the positive association between sTREM2 and Aβ_x-40_ abundance in CSF (Fig. 3). Replication of the sTREM2*rs1582763_A interaction effect using mass spectrometry measurement of Aβ_1-40_ peptide in ADNI yielded significant results (Table 4). Non-carriers of this allele exhibited decreased memory performance with higher CSF sTREM2 concentration while in minor allele carriers, cognition did not correspond to sTREM2 concentration (Supplemental Fig. 1).

Perhaps given directional associations with cognition and AD protection this protective variant decouples sTREM2 from an otherwise pathological process involving soluble Aβ abundance. For example, soluble, low-molecular-weight Aβ oligomers may contribute to synapse loss preceding neuronal death [36]. Aβ peptides are composed of 36–43 amino acids and are the various cleavage products of their transmembrane parent amyloid precursor protein (APP). While full-length Aβ_1-42_ displays rapid oligomerization and toxicity through the formation of insoluble fibrils [37], there are a number of studies showing neurotoxic effects of shorter Aβ peptide sequences (*i.e.*, 1–38, 1–40, N-terminal truncated) impacting synapse function and cognition in mice [38–40]. And notably, Aβ_1-38_ truncated at its C-terminal moiety, was identified as the second most abundant form after Aβ_1-40_ [38]; the latter makes up about 60% of Aβ species present in CSF [41]. Therefore, genetic regulation of the *MS4A* locus modulates the relationship between sTREM2 levels and the most abundant Aβ species in CSF. The lack of significant results with biomarkers of plaque (Aβ_1-42_ and the Aβ_1-42_ / Aβ_1-40_ ratio Supplemental Table 13) may point towards a more nuanced relationship between homeostasis of soluble amyloid species, TREM2 and MS4A. In risk variant carriers, sTREM2 levels are tightly coupled to soluble Aβ, while in protective variant carriers, sTREM2 levels decouple from soluble Aβ which may reflect a protective neuroinflammatory response to pathology.

The interpretation of the interaction between risk SNP rs6591561_G and CSF sTREM2 levels on Aβ_1-40_ in ADNI (Table 5 and Fig. 6A) should be interpreted cautiously as it was not originally observed in the VMAP discovery cohort. This may be due to a much smaller sample size (*N* = 127) compared to ADNI (*N* = 399) or otherwise due to differences in demographics between the two cohorts. This includes notable differences in average CSF sTREM2 expression across diagnoses as well as the prevalence of *APOE-ε4* carriers.

### *MS4A* protective variant drives the association between CSF sTREM2 and BBB permeability

Genetic regulation by protective rs1582763_A enhanced the positive association between sTREM2 and the CSF/plasma albumin ratio (Fig. 4). The positive relationship between sTREM2 and increased BBB permeability in carriers, but not non-carriers of AD-protective rs1582763_A may be important for a neuroinflammatory response to early changes in Aβ dysregulation which involves peripheral-central communication. The lack of sTREM2 coupling to the CSF/plasma albumin ratio in non-carriers may therefore reflect a decreased capacity to mount a proper neuroinflammatory response to early changes in the neuropathological landscape. This is supported by evidence showing CSF sTREM2 levels are normalized (reduced) during neuroinflammation by natalizumab treatment which targets and attenuates leukocyte trafficking across the BBB in patients with multiple sclerosis [42]. However, in AD, early neuroinflammation (and microglial response) may be beneficial to resolving neuropathology. Therefore, it is possible that in early stages of Aβ development that peripheral immune involvement may help augment sTREM2 levels in brain or vice versa. sTREM2 has been thought to diffuse to some extent through blood vessels reaching the brain parenchyma [43]. However, peripheral immune cells also express TREM2 and may contribute to the CSF reservoir of sTREM2 concentration. For example, infiltrating T cells also express TREM2 receptor which is attuned to their activation and helps modulate their inflammatory signaling but may also undergo proteolytic cleavage in the brain [44].

Alternatively, the coupling of increased CSF sTREM2 and CSF/plasma albumin ratio may be indicative of increased permeability of the choroid plexus (blood-CSF barrier) to solutes which is known to occur in AD [45], and subsequently, the increased accumulation of sTREM2 in CSF which had originated in plasma having been cleaved in peripheral tissues. Regardless of the origin of increases in CSF sTREM2, this positive association between CSF sTREM2 and the CSF/plasma albumin ratio occurs selectively in rs1582763_A carriers begging the question and extent of MS4A protein involvement in the BBB and or blood-CSF barrier regulation. The lack of CSF/plasma albumin measurement in ADNI precluded independent replication of this interaction result. Nonetheless, the intersection between barrier integrity, MS4A, and TREM2 proteins remains an uncharted topic which may shed light on early pathophysiological changes in AD and give rise to novel immunomodulatory therapeutic targets.

### *MS4A* genetic variation does not modulate the association between CSF sTREM2 and tau

There was no evidence of *MS4A* genotypes significantly interacting with sTREM2 levels on biomarkers of tau pathology. This observation suggests that genetic increases in sTREM2 levels through this locus near *MS4A4A* likely involve amyloid and BBB regulation but leaves out the very robust and consistent associations we see of CSF sTREM2 and t-tau and p-tau. This is interesting considering increases in CSF sTREM2 have historically been thought to represent a microglial response to neurodegeneration and yet genetic regulation by *MS4A*-AD associated SNPs did not modify this relationship in our analyses. It is possible that the interaction of MS4A and TREM2 proteins at the onset of amyloid seeding is of particular importance to the regulation of Aβ pathology and thus AD risk.

### *MS4A* risk variant attenuates the association between TREM2 transcript and Aβ1-38

Interestingly, rs6591561_G risk variant interacted with *TREM2* transcript on Aβ_1-38_ peptide levels measured using RNA sequencing and mass spectrometry at autopsy (Table 7 and Fig. 7). Furthermore, there was also a lack of interaction with tau peptide that parallels the findings above from biomarker analyses (Supplemental Table 12). These results highlight a more generalized relationship between MS4A and the TREM2 pathway beyond its known regulation of sTREM2 levels in CSF. Specifically, participants carrying the risk allele had lower levels of *TREM2* mRNA at autopsy and the relationship between increases in *TREM2* mRNA and increases in Aβ_1-38_ peptide was driven by non-carriers, suggesting this AD risk SNP may impair a potential cortical *TREM2* transcriptional response to amyloid (which has been previously characterized by this group using ROS/MAP data [46]). However, this finding necessitates replication in an independent cohort with available post-mortem data. The majority of sTREM2 protein in brain is thought to originate from TREM2 cleavage, although 25% is thought to originate from isoform specific generation [23]. Therefore, it is not entirely unexpected to find a lack of concordance between transcript and CSF results herein. These *MS4A* variant associations and interactions with transcript in the brain suggest that *MS4A* genetic variation may affect *TREM2* expression, however, further characterization of these variants on *TREM2* transcript expression, protein abundance, and post-translational modifications will be needed to complete the picture of how these genes intersect and contribute to AD pathogenesis.

### Strengths and weaknesses

There are several strengths to this study, including the utilization of multiple independent cohorts of aging and Alzheimer’s disease yielding large sample sizes and utilization of several highly sensitive methods of proteomic analysis. Independent replication of interactions using ADNI data increased confidence in these results. However, majority non-Hispanic White data limits genetic diversity making it hard to generalize findings to more diverse populations. This is especially pertinent given the recent finding that on average, African American, compared to non-Hispanic White individuals, have lower levels of CSF sTREM2 which has been attributed to an increased likelihood of carrying *TREM2* coding variants associated with decreased levels of sTREM2 and a lowered likelihood of carrying *MS4A* AD-associated rs1582763_A [47].

Furthermore, there were notable differences in the methods of CSF analyte measurement as well as participant demographics between VMAP and ADNI that could have contributed to inconsistent findings herein. The relatively small sample size in VMAP could also contribute to the inconsistent results with rs6591561_G. It is also possible that heterogeneity in production and clearance rates may impact results as individuals may have notable baseline differences in CSF/ISF flow of soluble Aβ protein abundance. Additionally, functional studies are needed to validate the association with the CSF/plasma albumin ratio. Future work aims to address potential interactions of MS4A and TREM2 pathways at further time points in dementia progression and with respect to the outcomes on diverse concomitant neuropathology such as vascular comorbidities.

In summary, using VMAP as a discovery cohort, we find evidence that genetic regulation at the *MS4A* locus may confer protection against AD at least in part via augmentation of the TREM2 pathway, possibly increases in sTREM2 CSF reservoir in response to soluble Aβ pathology, replicating results in an independent dataset (*N* = 399) in ADNI. We also find evidence that this regulation may confer AD risk via a proposed dampening of *TREM2* transcriptional response to Aβ using available autopsy data in ROS/MAP. The present results indicate that the coupling between TREM2 and these shorter Aβ species lies at the intersection of the complex interplay between *MS4A* genetic risk and TREM2 biology.

### Supplementary Information

**Supplementary Material 1.**

## Data Availability

VMAP data can be requested within our data sharing portal and will be made freely available to qualified investigators (https://www.vmacdata.org/vmap/data-requests).
