# Supplementary material for: Variants in the MS4A cluster interact with soluble TREM2 expression on biomarkers of neuropathology
Source: Mol Neurodegener. 2024 May 18;19:41. doi: 10.1186/s13024-024-00727-7 (PMC11101336; doi:10.1186/s13024-024-00727-7)
Supplement: Supplementary file 1 — Supplementary Material 1. [file 13024_2024_727_MOESM1_ESM.docx]

Supplemental Tables

**Supplemental Table 1. VMAP commercially available kit information**

| CSF Biomarker | Assay Kit Name | Company | Catalogue Number |
| --- | --- | --- | --- |
| Aβx-40 | MSD® Aβ Triplex Assay | Meso Scale Discovery, Rockville, MD | K15148 |
| Aβx-42 | MSD® Aβ Triplex Assay | Meso Scale Discovery, Rockville, MD | K15148 |
| Aβ1-42 | INNOTEST® β-Amyloid (1-42) | Fujirebio, Ghent, Belgium | 81583 |
| p-tau_181_ | INNOTEST® PHOSPHO-Tau (181P) | Fujirebio, Ghent, Belgium | 81581 |
| t-tau | INNOTEST® hTau Ag | Fujirebio, Ghent, Belgium | 81579 |
| NfL | NF-light™ ELISA RUO | UmanDiagnostics, Umeå, Sweden | 10-7002 RUO |
| albumin | IMMAGE®  Immunochemistry System | Beckman Coulter, Brea, CA | 447600 |

**Supplemental Table 2. ADNI Cohort Demographics**

| Characteristic | Clinical Diagnosis | | | Total  (N=440) |
| --- | --- | --- | --- | --- |
|  | Normal Cognition (N=138) | Mild Cognitive Impairment (N=270) | Alzheimer’s Disease (N=32) |  |
| Male, no. (%) | 72 (52) | 153 (57) | 20 (63) | 245 (56) |
| Age (baseline) | 74±6.03 | 71±7.38 | 76±10.26 | 73±7.39 |
| Education | 17±2.60 | 16±2.67 | 16±2.64 | 16±2.66 |
| sTREM2 CSF pg/mL | 4108±2034.21 | 3933±1968.17 | 4900±2619.85 | 4058±2052.24 |
| *APOE-*ε4 carriers, no. (%) | 35 (25) | 125 (46) | 22 (69) | 182 (41) |

Values are presented as mean±standard deviation, unless otherwise indicated.

| Characteristic | Clinical Diagnosis | | | Total  (N=577) |
| --- | --- | --- | --- | --- |
|  | Normal Cognition (N=188) | Mild Cognitive Impairment (N=168) | Alzheimer’s Disease (N=221) |  |
| Male, no. (%) | 67 (36) | 54 (32) | 71 (32) | 192 (33) |
| Age (at death) | 88±6.45 | 90±6.25 | 90±6.41 | 89±6.46 |
| Education (years) | 16±3.64 | 16±3.22 | 16±3.48 | 16±3.46 |
| *APOE-*ε4 carriers, no. (%) | 32 (17) | 27 (16) | 81 (37) | 140 (24) |

**Supplemental Table 3. ROS/MAP Cohort Demographics**

Values are presented as mean±standard deviation, unless otherwise indicated. Cohort is non-Hispanic White.

**Supplemental Table 4. sTREM2 * Presence of *MS4A* SNP Interaction on CSF AD biomarkers excluding subjects with probable CAA**

| VMAP Outcome | *rs1582763* | | *rs6591561* | |
| --- | --- | --- | --- | --- |
|  | β | P Value | β | P Value |
| Aβ_x-42_ | -0.095 | **0.005*** | 0.015 | 0.602 |
| CSF/plasma albumin ratio | 0.001 | **0.003*** | -0.0002 | 0.338 |
| Aβ_x-40_ | -0.498 | **0.006*** | 0.182 | 0.236 |
| t-Tau | -0.041 | 0.064 | 0.006 | 0.740 |
| Aβ_1-42_ | -0.051 | 0.095 | -2.122 | 0.674 |
| Tau_181P_ | -0.005 | 0.055 | 0.001 | 0.663 |

Cross-sectional linear regression models assessed interactions of sTREM2 CSF protein levels and the presence of *MS4A* variant, rs1582763, on VMAP CSF AD biomarker outcomes as quantified by ELISA.

Bold represents statistical significance set to a *priori* threshold P < 0.05

***** Signifies significance after multiple corrections using the Benjamini & Hochberg (1995) false discovery rate based on number of tests completed

**Supplemental Table 5. ADNI sTREM2 * Presence of *MS4A* SNP Interaction on CSF AD biomarkers excluding subjects with *MS4A* rare variants**

| ADNI Outcome | *rs1582763* | | *rs6591561* | |
| --- | --- | --- | --- | --- |
|  | β | P Value | β | P Value |
| Aβ_1-40_ | -0.157 | 0.218 | 0.268 | **0.018*** |
| Aβ_1-38_ | -0.026 | 0.391 | 0.056 | **0.037*** |
| Aβ_1-42_ | -0.011 | 0.760 | 0.035 | 0.261 |

Bold represents statistical significance set to a *priori* threshold P < 0.05

***** Signifies significance after multiple corrections using the Benjamini & Hochberg (1995) false discovery rate based on the number of tests completed

**Supplemental Table 6. ROS/MAP Interaction Effects of *TREM2* mRNA*SNP on Aβ Neuropathology excluding subjects with *MS4A* rare variants**

| Neuropathology Outcome Measure | *rs1582763* | | *rs6591561* | |
| --- | --- | --- | --- | --- |
|  | Estimate | P.int.Value | Estimate | P.int.Value |
| Aβ_1-38_ (SRM) | 0.160 | 0.094 | -0.272 | **0.004*** |
| Aβ (total) (SRM) | -0.070 | 0.838 | 0.190 | 0.569 |

Cross-sectional linear regression models assessed interactions of *TREM2* mRNA levels and the presence of *MS4A* variants, on ROS/MAP Aβ neuropathology as quantified by SRM proteomic analysis.

Bold represents statistical significance set to a *priori* threshold P < 0.05

* Signifies significance after multiple corrections using the Benjamini & Hochberg (1995) false discovery rate based on the number of tests completed

**Supplemental Table 7. Carriers of rare *MS4A* variants by cohort**

|  | VMAP | ADNI | ROSMAP |
| --- | --- | --- | --- |
| R47H | 0 | 0 | 0 |
| R62H | 0 | 12 | 7 |
| D87N | 0 | 4 | 4 |
| H157Y | 0 | 0 | 1 |

All carriers have one copy of the minor allele

**Supplemental Table 8. Carriers of *MS4A* variants by cohort**

|  | VMAP  (N = 127) | | ADNI  (N = 399) | |
| --- | --- | --- | --- | --- |
|  | Carriers | Non-carriers | Carriers | Non-carriers |
| rs1582763  Minor Allele (A<G) | 79 | 48 | 239 | 160 |
| rs6591561  Minor Allele (G<A) | 66 | 61 | 212 | 187 |

**Supplemental Table 9. Main Effects of *MS4A* SNPs on CSF AD Biomarkers in VMAP**

| VMAP Outcome | *rs1582763* | | *rs6591561* | |
| --- | --- | --- | --- | --- |
|  | Estimate | P Value | Estimate | P Value |
| Aβ_x-42_ | -3.847 | 0.940 | 6.469 | 0.894 |
| CSF/plasma albumin ratio | 0.210 | 0.633 | 0.125 | 0.763 |
| Aβ_x-40_ | -154.400 | 0.624 | 324.414 | 0.274 |
| t-Tau | -32.034 | 0.389 | 27.355 | 0.436 |
| Aβ_1-42_ | 29.199 | 0.526 | -10.315 | 0.812 |
| Tau_181P_ | -4.806 | 0.278 | 3.115 | 0.457 |

Bold represents statistical significance set to a *priori* threshold P < 0.05

***** Signifies significance after multiple corrections using the Benjamini & Hochberg (1995) false discovery rate based on number of tests completed

**Supplemental Table 10. Main Effects of *MS4A SNPs* on CSF AD Biomarkers in ADNI**

| ADNI Outcome | *rs1582763* | | *rs6591561* | |
| --- | --- | --- | --- | --- |
|  | β | P | β | P |
| Aβ_1-40_ | -14.573 | 0.935 | 376.070 | **0.049*** |
| Aβ_1-38_ | -20.506 | 0.627 | 94.012 | **0.036*** |
| Aβ_1-42_ | -45.528 | 0.323 | 135.412 | **0.006*** |

Bold represents statistical significance set to a *priori* threshold P < 0.05

***** Signifies significance after multiple corrections using the Benjamini & Hochberg (1995) false discovery rate based on number of tests completed

**Supplemental Table 11. Main Effects of *TREM2* mRNA on Tau Neuropathology**

| Neuropathology Outcome Measure | Estimate | Std. Error | P Value |
| --- | --- | --- | --- |
| Tau AT8 (SRM) | 0.025 | 0.013 | 0.054 |

Bold represents statistical significance set to a *priori* threshold P < 0.05

* Signifies significance after multiple corrections using the Benjamini & Hochberg (1995) false discovery rate based on number of tests completed

**Supplemental Table 12. Interaction Effects of *TREM2* mRNA*SNP on Tau Neuropathology**

| Neuropathology Outcome Measure | *rs1582763* | | | *rs6591561* | | |
| --- | --- | --- | --- | --- | --- | --- |
|  | Estimate | Std. Error | P.int.Value | Estimate | Std. Error | P.int.Value |
| Tau AT8 (SRM) | -0.038 | 0.026 | 0.154 | 0.038 | 0.026 | 0.151 |

Bold represents statistical significance set to a *priori* threshold P < 0.05

* Signifies significance after multiple corrections using the Benjamini & Hochberg (1995) false discovery rate based on number of tests completed

**Supplemental Table 13. Interaction Effects of CSF *TREM2**SNP on Aβ42/40 Ratio in VMAP**

| Neuropathology Outcome Measure | *rs1582763* | | *rs6591561* | |
| --- | --- | --- | --- | --- |
|  | β | P | β | P |
| Aβ42/40 ratio | -7.11e-05 | 0.078 | -2.598e-05 | 0.411 |
